# Effects of Al Doping on the Properties of ZnO Thin Films Deposited by Atomic Layer Deposition

**DOI:** 10.1186/s11671-016-1625-0

**Published:** 2016-09-17

**Authors:** Chen-Hui Zhai, Rong-Jun Zhang, Xin Chen, Yu-Xiang Zheng, Song-You Wang, Juan Liu, Ning Dai, Liang-Yao Chen

**Affiliations:** 1 Department of Optical Science and Engineering, Ministry of Education, Key Laboratory of Micro and Nano Photonic Structures, Fudan University, 220 Handan Road, Shanghai, 200433 China; 2 National Laboratory for Infrared Physics, Shanghai Institute of Technical Physics, Chinese Academy of Sciences, Shanghai, 200083 China; 3 School of Optoelectronics, Beijing Institute of Technology, Beijing, 100081 China

**Keywords:** Al-doped ZnO thin films, Atomic layer deposition, Optical properties, Spectroscopic ellipsometry, Electrical properties

## Abstract

The tuning of structural, optical, and electrical properties of Al-doped ZnO films deposited by atomic layer deposition technique is reported in this work. With the increasing Al doping level, the evolution from (002) to (100) diffraction peaks indicates the change in growth mode of ZnO films. Spectroscopic ellipsometry has been applied to study the thickness, optical constants, and band gap of AZO films. Due to the increasing carrier concentration after Al doping, a blue shift of band gap and absorption edge can be observed, which can be interpreted by Burstein-Moss effect. The carrier concentration and resistivity are found to vary significantly among different doping concentration, and the optimum value is also discussed. The modulations and improvements of properties are important for Al-doped ZnO films to apply as transparent conductor in various applications.

**Electronic supplementary material:**

The online version of this article (doi:10.1186/s11671-016-1625-0) contains supplementary material, which is available to authorized users.

## Background

A transparent conductive oxide (TCO) has received considerable attentions and been widely used in electronic and optoelectronic devices [1], such as solar cells [2], liquid crystal [3], and high-definition displays [4], due to their low resistivity and high transmittance. There are various TCO materials, including In, Sb, Zn, Cd, Sn metal oxides, and their composite oxides. Among them, indium tin oxide (ITO) film is the most widely used TCO material [5]. However, the scarce and toxic nature of indium and instability of ITO are the main obstacles for its further development, which arouses the interests of researchers to explore alternative TCO materials for ITO. As a candidate for TCO, ZnO films doped with trivalent metal cations have attracted considerable attentions [6–9]. Thereinto, Al-doped ZnO (AZO) film is one of the most promising candidates [10], since it has many advantages, such as low cost, abundant resource, non-toxicity, and good stability in hydrogen plasma. Importantly, the optical and electrical behaviors of AZO films can be improved or modified by controlling their doping level [11], which is critical to achieving functionalization and tunability of TCO-based devices. Therefore, it is useful to investigate the correlation between the properties of AZO films and the concentration of Al doping.

Various methods have been used to prepare AZO films, including atomic layer deposition (ALD) [12, 13], chemical vapor deposition (CVD) [14, 15], magnetron sputtering [16, 17], and pulsed laser deposition (PLD) [18]. Comparing to other techniques, ALD is an excellent deposition technique based on self-limiting surface chemical reactions that can be used to prepare highly uniform and smooth films while their thickness can be precisely controlled.

Various properties of ALD-based AZO films have been reported by many research groups [19–22]. Among these properties, optical properties are generally studied and analyzed based on transmission and photoluminescence spectra [21, 22]. However, there are few reports on the properties evaluated by spectroscopic ellipsometry (SE) analysis [17]. SE known for its precision and non-destructiveness is a useful tool for the optical characterization of nanostructures [23, 24]. Thickness, optical constants, and band gap energy information can be determined accurately by using SE. With increasing the Al doping level, the modulation of the optical properties provides references to the change of electrical characteristics.

In this work, we investigated the structural, optical, and electrical properties of AZO thin films deposited by ALD technique with a wide range of doping levels. The effects of concentration of Al doping on properties of AZO thin films were discussed in detail. The thickness, optical constants, and band gap of AZO samples were calculated by fitting ellipsometry data in a broadband spectral region. The modulated transmittance of AZO thin films was shown by transmission spectrum measurement. The blue shift of band gap and absorption edge were observed and discussed in light of Burstein-Moss effect. The electrical properties of the films were measured by using a Hall effect measurement system. The optimum concentration of Al for resistivity was studied with the structural and optical properties keeping excellent as well.

## Methods

**Fig. 1:**
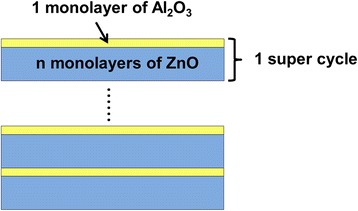
The structure diagram of the AZO films

Both pure ZnO and Al-doped ZnO thin films were prepared on the Si and quartz substrates through a custom-made ALD reactor. The deposition procedure was at a temperature of 150 °C and a working pressure of 80 Pa. Diethylzinc [DEZ; Zn(C_2_H_5_)_2_] and trimethylaluminum [TMA; Al(CH_3_)_3_] were used as the precursors for Zn and Al, respectively, and deionized water (H_2_O) was used as the oxidant reactant. High purity nitrogen (N_2_) with a gas flow rate of 50 sccm (standard cubic centimeters per minute) was used as the carrier to deliver precursors into the chamber and purging gas to take the needless products away from the chamber. During the deposition process, the DEZ and H_2_O were alternatively introduced into the chamber to grow the ZnO films through DEZ-H_2_O cycles (DEZ/exposure/N_2_/H_2_O/exposure/N_2_), with pulse times of 0.03/3/15/0.03/5/15 s. For Al-doping into the ZnO films, TMA-H_2_O cycles of Al_2_O_3_ were introduced with the same process as the DEZ-H_2_O. The ZnO and Al_2_O_3_ monolayers grow through their surface reactions [25]. The structure diagram of the investigated AZO film is shown in Fig. 1. The film deposition consists of several super cycles while one super cycle consists of one monolayer of Al_2_O_3_ and *n* monolayers of ZnO. Different Al content of AZO films was obtained by varying the number of ZnO monolayers during one super cycle. Hence, various numbers of DEZ-H_2_O cycles and one TMA-H_2_O cycle were repeatedly carried out for film depositions, as the cycle ratio given in Table 1. Same number of ZnO monolayers (N2) was adopted for each sample to fix the thickness of the main body of the films which could avoid the thickness effects on the properties of samples. According to N2 and the cycle ratio of DEZ-H_2_O and TMA-H_2_O, the number of super cycle or Al_2_O_3_ monolayers (N1) and the total numbers of cycles (*N*, *N* = N1 + N2) is also determined.

**Table 1:** Deposition parameters for samples, Al composition of AZO films via XPS, and film thicknesses fitted by SE

Samples	DEZ-H_2_O/TMA-H_2_O cycle ratio	*N*1	*N*2	*N*	at. %Al	Thicknesses (nm)
ZnO	–	–	200	200	–	41.2
AZO 50:1	50:1	4	200	204	3.7 %	41.7
AZO 20:1	20:1	10	200	210	4.9 %	44.3
AZO 10:1	10:1	20	200	220	7.1 %	46.8
AZO 5:1	5:1	40	200	240	12.7 %	48.6

The chemical compositions and chemical bond states of the thin films were characterized using X-ray photoelectron spectroscopy (XPS). XPS analysis was carried out through a scientific spectrometer (Axis Ultra DLD) with an Al KR X-ray source (1486.6 eV). The phase and crystallinity of samples were measured by X-ray diffraction (XRD; Bruker D8 ADVANCE) with Cu Kα radiation (*λ* = 1.5418 Å). The images of atomic force microscopy (AFM) of samples were obtained by using a Bruker Dimension Icon microscope VT-1000 System operated in tapping mode. The spectroscopic ellipsometry measurement was carried out by a vertical variable-angle SE (V-VASE; J.A. Woollam Co., Inc.) in the wavelength range of 200–1000 nm with a spectral resolution of 5 nm. The incident angle was selected as 65, 70, and 75° to insure the reliability of fitting results. Optical transmission spectra were measured with a double beam UV-VIS-NIR spectrophotometer (Shimadzu UV-3600) in the wavelength range of 250–1000 nm. And the electrical properties of the films were measured by using thevan der Pauw method with a Hall effect measurement system (Ecopia HMS3000). All these measurements were carried out at room temperature.

## Results and Discussion

### Composition and Structure Analysis

**Fig. 2:**
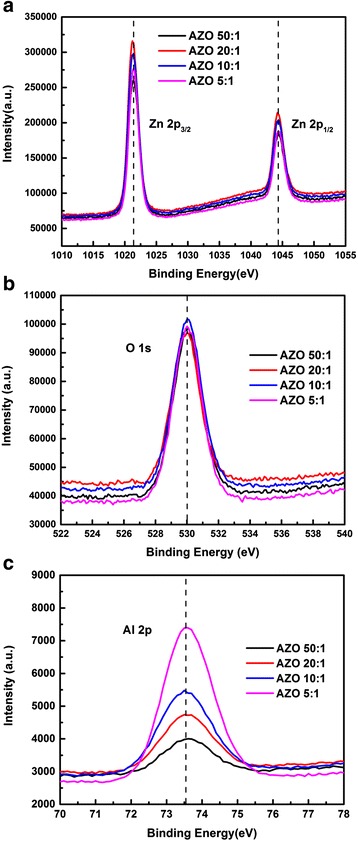
XPS spectra of AZO films grown on Si substrates: (**a**) Zn 2p peaks; (**b**) O 1s peaks; (**c**) Al 2p peaks

To verify the concentration of Al doping, the XPS measurement was carried out on Al-doped ZnO films. Figure 2 reveals the XPS spectra of the AZO films after calibration with carbon peak. In Fig. 2a, the high symmetry energy peak of Zn 2p_3/2_ is located at 1021.3 ± 0.1 eV, which is approximately equal to the value of Zn in bulk ZnO [26]. It indicates that Zn in AZO films exists in the oxidized states. From Fig. 2b, the main peak located at 530.0 ± 0.1 eV is assigned to lattice oxygen bonded as O^2−^ ions in the ZnO matrix [27]. Here, carbon composition has been removed from those spectra, so no peak of carbon composition can be observed in Fig. 2b. As for Fig. 2c, the energy peaks of Al 2p exhibit a symmetry feature, and is located at around 73.5 ± 0.1 eV which is corresponding to the characteristic peak of Al_2_O_3_ [28]. The growth of peak intensity indicates the increase of Al concentration in the films. The concentration of Al is calculated from the ratio of Zn, Al, and O atoms, as shown in Table 1. With varying the cycle ratio of DEZ-H_2_O and TMA-H_2_O from 50:1 to 5:1, the Al concentration increases from 3.7 to 12.7 %.

**Fig. 3:**
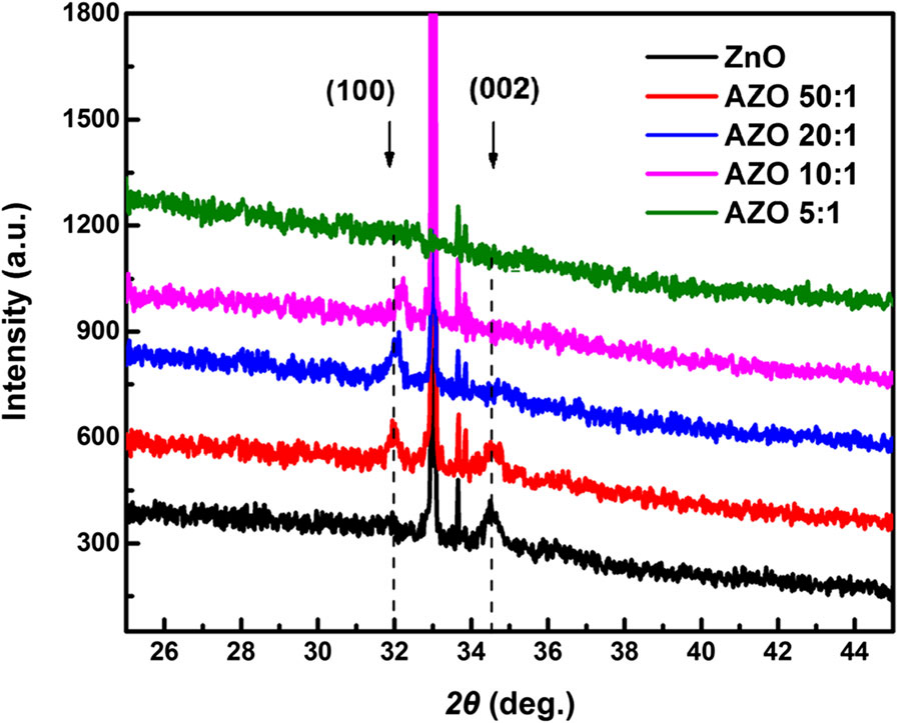
XRD patterns of samples grown on Si substrates after annealing

The X-ray diffraction patterns of samples grown on Si are shown in Fig. 3. To obtain clearer phenomenon of crystallinity, the samples were annealed at 400 °C in N_2_ atmosphere for 1 h. The crystalline structure of the films exhibits a hexagonal wurtzite structure with growth directions of ZnO (100) or (002). No clear peaks of Al_2_O_3_ manifests the deposited Al_2_O_3_ is amorphous under the growth conditions in this work. The crystalline state and crystal orientation of these films are found to change with the increasing doping concentration of Al^3+^. The pure ZnO film shows a preferred growth with ZnO (002) direction, and the peak position locates at about 34.5°. However, the (002) peak disappears and new ZnO (100) peak becomes dominant after Al doping. The evolution of ZnO peaks demonstrates that Al-doping affects the growth mode of ZnO films, which is similar to the results reported by Banerjee et al. [29]. With further increasing Al doping, no peak is observed for AZO 5:1 sample, which indicates the AZO film at 12.7 at.% Al is amorphous. Since the Al_2_O_3_ layer by ALD is amorphous in our growth conditions, the Al_2_O_3_ doping layers destroy the crystal quality of the AZO 5:1 films, which causes the disappearance of its (100) peak. From Fig. 3, it can also be observed that the ZnO (100) peak shifts to a larger diffraction angle with the increasing Al^3+^ content. Here, the Al^3+^ ions (ion radius 0.53 Å) is smaller than Zn^2+^ ions (ion radius 0.74 Å), so the increasing Al concentration will reduce the lattice constant of samples by substitutions of Zn^2+^ ions with Al^3+^ [30].

**Fig. 4:**
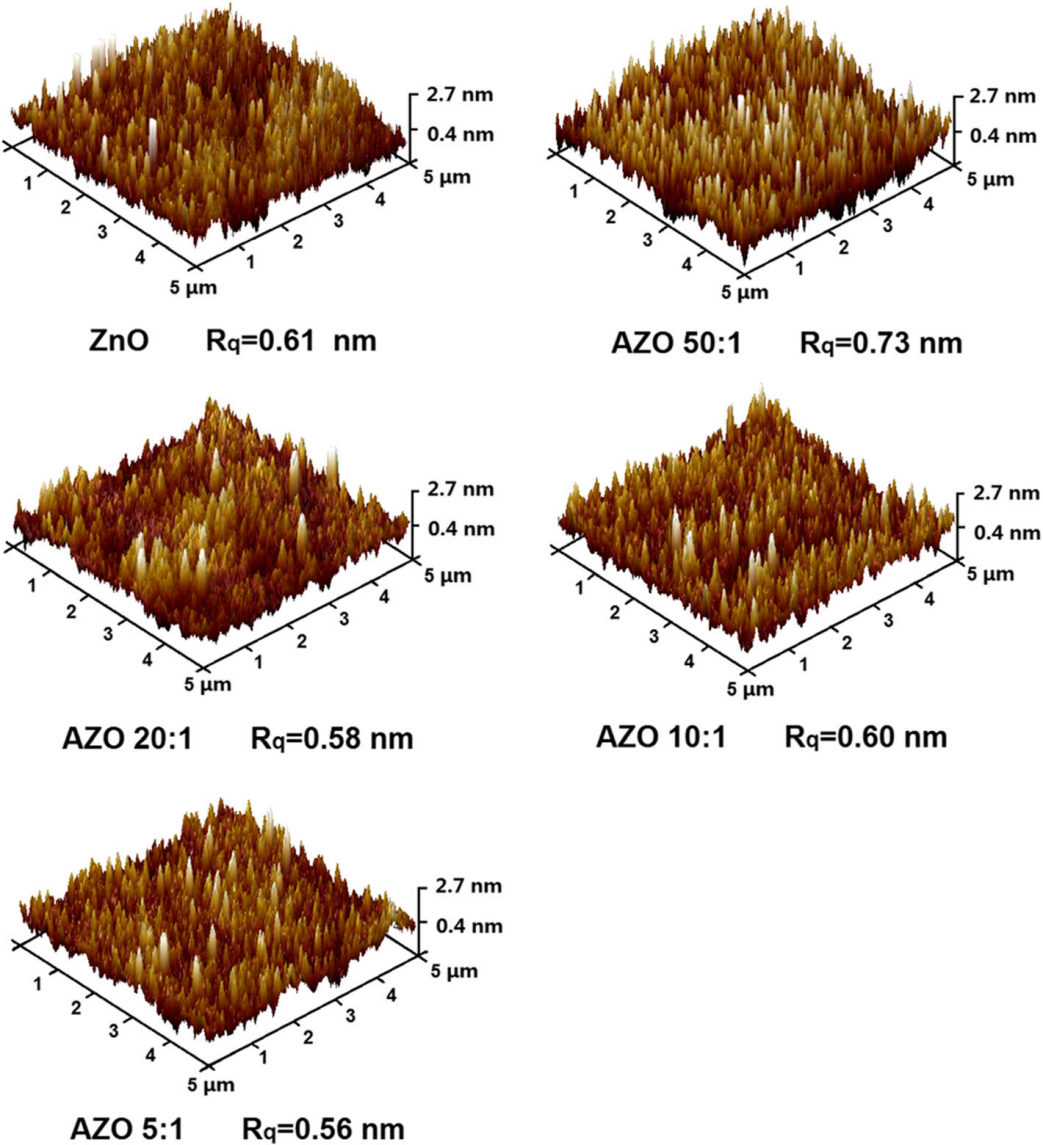
AFM 3D images of pure ZnO and AZO thin films grown on Si substrates

The surface morphologies of the samples grown on Si substrates are analyzed by using the AFM method with a scanning area of 5 × 5 μm^2^. The 3D AFM images of the samples are shown in Fig. 4. All the films show good uniformity over the whole scanning area. The hill-shaped features with average lateral dimensions 50–100 nm can be observed on the surface of samples. The root-mean-square roughness (*R*_*q*_) of each samples calculated from AFM data is less than 1.00 nm, as given under their AFM images, respectively. It is clear that all samples present smooth surface with inessential surface roughness. Therefore, the surface scattering is weak enough for SE analysis, which makes the analysis more reliable [31].

### Optical Properties of Samples

Spectroscopic ellipsometry is generally applied for the investigation of the thickness, optical properties of samples, which is based on measuring the change in the polarization state of a linearly polarized light reflected from the sample surface. In order to get more accurate information, Si is selected as the substrate to provide enough reflected light in SE measurement. For SE analysis, an optical model of our samples is firstly constructed, which consists of a semi-infinite Si substrate/pure ZnO or AZO film/air ambient structure. No roughness layer is introduced, due to the smooth surface of samples revealed by AFM measurements. The obtained ellipsometry spectra (Ψ and Δ at range of 200–1000 nm) of the films are fitted by using the Forouhi-Bloomer dispersion model (Additional file 1)[32]. This dispersion model is widely used to describe the optical properties in the spectral region which is dominated by inter-band transitions and contains the information of the band gap [32]. The thickness, optical constants, and band gap of the films are evaluated in a fitting procedure by minimizing the root-mean-square error (RMSE) which defined as follows [33]:

1 $$ \mathrm{RMSE}=\sqrt{\frac{1}{2x-y-1}{\displaystyle {\sum}_{i=1}^x\left[{\left({\varPsi}_i^{\mathrm{cal}}-{\varPsi}_i^{\exp}\right)}^2+{\left({\varDelta}_i^{\mathrm{cal}}-{\varDelta}_i^{\exp}\right)}^2\right]}} $$

Here, *x* is the number of data points in the spectrums, *y* is the number of variable parameters in the model, and “exp” and “cal” represent the experimental and the calculated data, respectively.

**Fig. 5:**
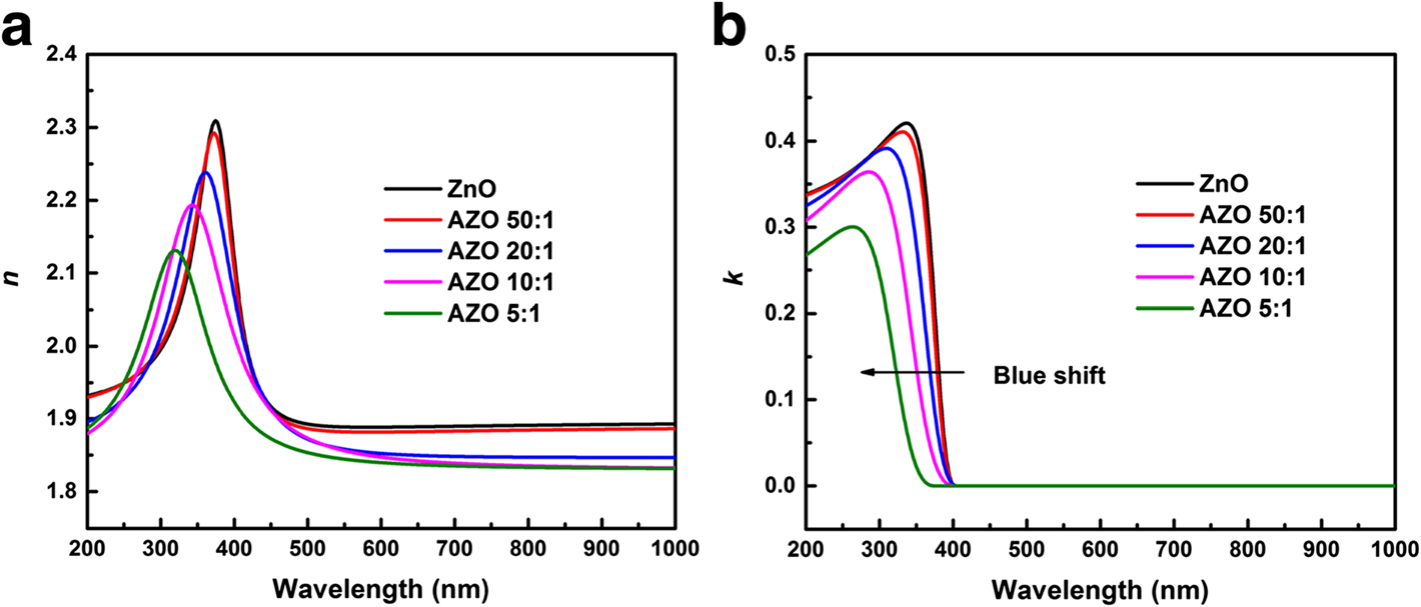
The optical constants of samples grown on Si substrates: **a** the refractive index *n*. **b** The extinction coefficient *k*

The fitted thickness of samples is shown in Table 1. With the increasing concentration of Al, the thickness of the film displays a growing trend due to the increased total numbers of cycles. Figure 5 illustrates the refractive index *n* and extinction coefficient *k* of the films with various doping levels at the wavelength range of 200–1000 nm. The calculated optical constants of the pure ZnO films obtained by ALD are in good agreement with our previous work [34]. Due to the Al doping is not deep enough, optical constants of AZO 50:1 is very close to pure ZnO. Figure 5a demonstrates the refractive index *n* of the films with various doping levels. Obviously, the refractive index of films decreases gradually with doping level increasing, since Al impurity can act as effective *n*-type donors to generate free carriers. The doping of Al_2_O_3_ increases the free carrier concentration in films, which results in the decrease of the refractive index of the films [35]. So, the refractive index can be modulated by Al doping level. Figure 5b describes the extinction coefficient *k* of the films with various doping levels. It can be seen that the *k* of all films is close to zero infinitely in the wavelength range of 400–1000 nm, which indicates that the films are nearly transparent in this wavelength region. Besides, a blue shift of the absorption edge can be observed with the increasing doping level.

**Fig. 6:**
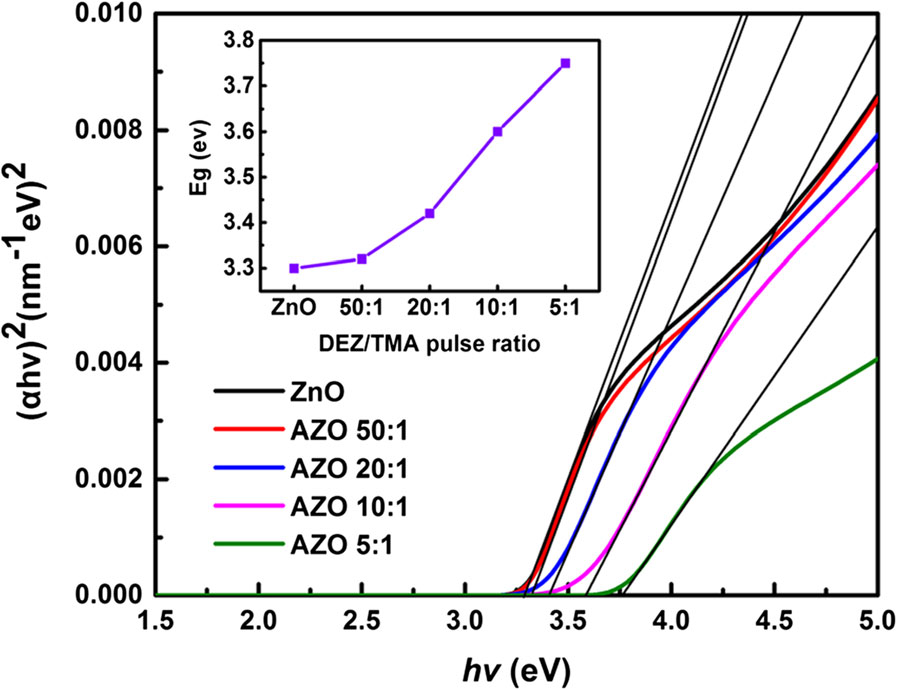
Evaluated optical band gap of samples grown on Si substrates

In order to better understand the blue shift of absorption edge, Tauc method is used to calculate the band gap of the sample by using formulas as follows [35]:

2 $$ {\left(\alpha h\nu \right)}^2=A\left(E-{E}_g\right) $$

3 $$ \alpha =\frac{4\pi k}{\lambda } $$

where *A* is a constant, *E*_*g*_ is the optical band gap energy, *α* is the optical absorption coefficient which can be calculated from extinction coefficient (*k*) and wavelength of SE results. In order to simplify the calculation, Tauc extrapolation is utilized to obtain the band gap energy of samples. As shown in Fig. 6, we made a plot of (*αhν*)^2^ versus the photon energy. The optical band gap can be determined by the linear fitting. Through extrapolation, the point value of fitted line and *x* axis is *E*_*g*_, as revealed in the inset figure of Fig. 6. The band gap of the ZnO film is 3.3 eV, which agrees well with the ideal band gap of pure ZnO. And the *E*_*g*_ displays a growing trend with the increasing concentration of Al. The tendency is similar to that of other elements doped ZnO and In_2_O_3_:Sn films (TCO materials) [36]. It can be interpreted by the Burstein-Moss effect [36, 37]. ZnO is a *n* type semiconductor material with direct transition, and its Fermi level will enter into the conduction band when it is heavily doped. The state below Fermi level is occupied by electrons. The absorption transition process of light can only exist between the valence band and the vicinity to Fermi level. It results the optical band gap of films moves to the high energy region. Moreover, the Burstein-Moss effect is related to the carrier density. Extrinsic Al^3+^ are substituted for Zn^2+^ in the AZO films, so the spare electrons from Al^3+^ can increase the concentration of free carriers in films, resulting in the growth of optical band gap of the films. It can be described by the equation as follows [18]:

4 $$ {E}_g={E}_{g0}+\varDelta {E}_g^{\mathrm{BM}}={E}_{g0}+\frac{h^2}{8{m}_{\mathrm{e}}^{*}}{\left(\frac{3}{\pi}\right)}^{2/3}{n}_e^{2/3} $$

where *E*_*g*0_, ∆*E*_*g*_^BM^, *n*_*e*_, *h*, and *m*_e_^*^ are the intrinsic forbidden band width, band gap increment caused by Burstein-Moss effect, electron carrier density, Planck’s constant, and the effective electron mass in the conduction band, respectively. In addition, the ideal band gap of pure ZnO is 3.3 eV and that of Al_2_O_3_ is 8.7 eV [29]. So, with the increasing Al doping level, the band gaps of films are increased.

**Fig. 7:**
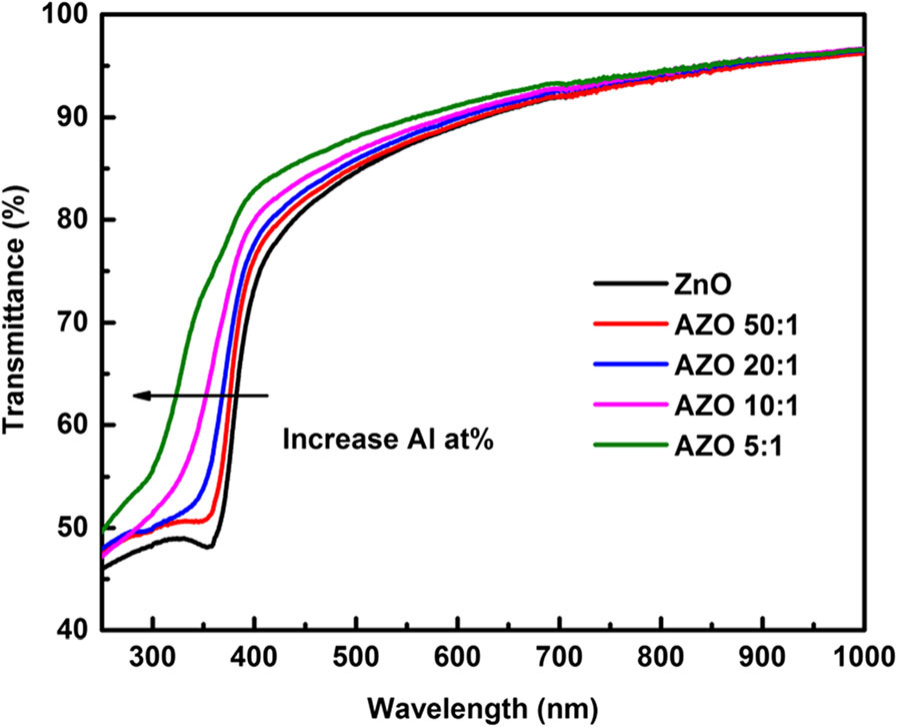
Transmittance spectra of samples grown on quartz substrates with various doping levels

Figure 7 illustrates the transmittance spectra of samples with various doping levels. The absorption edge of the samples can be found in the range from 300 to 400 nm. Blue shift of absorption edge is observed clearly with rising Al doping level, which is consistent to the results of SE analysis shown in Fig. 5b. This blue shift could be attributed to the rising of band gap energy resulting from Burstein-Moss effect. With the increasing Al doping level, the optical band gap of films is growing, which causes the absorption edge shift to short wavelength region. Moreover, it is clear that the optical transmittance of the films was slightly enhanced with the growing Al content, and the transmittance in the visible region is still higher than 85 %. The transmittance value is close to that of other common TCO films [38], which is important for the applications in the field of solar cell.

### Electrical Properties of Samples

**Fig. 8:**
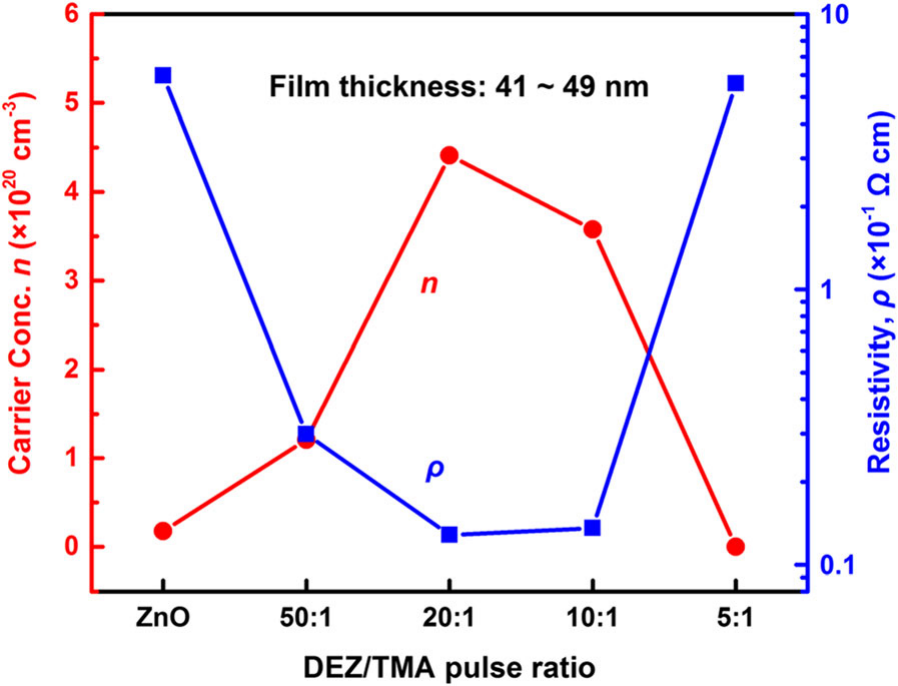
Carrier concentration and resistivity of samples grown on quartz substrates

Transparent conductor is one of the most common applications of AZO films. The electrical property of AZO films is also an important factor for its performance in devices. So, carrier concentration and resistivity of the films are assessed by using a Hall effect measurement system. In order to avoid the spatial resistivity distribution, the films are deposited on quart substrates, and their test results are revealed in Fig. 8. In the case of pure ZnO film, the carrier concentration and the resistivity were 1.77 × 10^19^ cm^−3^ and 5.98 × 10^−1^ Ωcm, respectively. As Al incorporated into ZnO films, the carrier concentration of AZO films at first increases rapidly and then reaches to the maximum value of 4.41 × 10^20^ cm^−3^ for AZO 20:1 film. After that, the carrier concentration of AZO films was abruptly reduced. As for the resistivity, it displays an opposite tendency, and the lowest value is 1.28 × 10^−2^ Ωcm also for AZO 20:1 film. The carrier concentration and resistivity of our AZO films are similar to other results of ALD-based AZO samples [20, 29, 39]. But it is slight different from those of AZO films deposited by the other methods, which use the bulk incorporation of dopants. By contrast, Al atoms in ALD-based AZO films would be more easily to cluster and form stoichiometric Al_2_O_3_ (insulator) [20]. Therefore, the carrier concentration of ALD-based AZO samples is a little lower, while the resistivity is a little higher.

From Fig. 8, the resistivity of pure ZnO is the highest due to its low carrier concentration. After Al doping, extrinsic Al^3+^ substitutes into host Zn^2+^ sites and provides an extra electron, which improves the carrier concentration. So, the resistivity decreases in the beginning. With the improving doping level, the increase of Al_2_O_3_ (insulator) concentration leads to the rise of resistivity. In addition, the lowest resistivity is 1.28 × 10^−2^ Ωcm for AZO 20:1 film, as shown in Fig. 8. So, the optimum Al concentration is 4.9 at.%, AZO 20:1, which is deposited by controlling a number of super cycles consistent with a DEZ/H_2_O:TMA/H_2_O cycle ratio of 20:1, as mentioned in the experimental section. Furthermore, AZ0 20:1 films possessed not only optimum carrier concentration and resistivity but also excellent optical transmission in solar cell absorption wavelength range. It is crucial for AZO films to be applied as transparent conductor.

## Conclusions

We have investigated the structural, optical, and electrical properties of ALD-based AZO films with doping concentrations ranging from 3.7 to 12.7 at.% for applications as transparent conductor materials. The inessential surface roughness within 1.00 nm shows the high morphological quality of the films deposited by ALD technology. The diffraction peak shifts from ZnO (002) to ZnO (100) with increasing Al content indicate that Al doping can change the growth mode of ZnO films. SE analysis has been adopted to reveal the thickness, optical constants, and band gap of samples. A blue shift of absorption edge of extinction coefficient appears with a growing trend of optical band gap energy due to Burstein-Moss effect. Meanwhile, the blue shift is also shown in optical transmission spectra with the average transmittance that is beyond 85 % in the visible region. Moreover, the lowest resistivity, 1.28 × 10^−2^ Ωcm, is found for AZO 20:1 film with an Al content of 4.9 at.%. The results of this study can be a useful reference for practical applications and engineering design.

## Additional file

Additional file 1: Supporting information. (DOCX 298 KB)

## Abbreviations

AFM: Atomic force microscopy
ALD: Atomic layer deposition
AZO: Al-doped ZnO
CVD: Chemical vapor deposition
DEZ: Diethylzinc
ITO: Indium tin oxide
PLD: Pulsed laser deposition
RMSE: Root-mean-square error
Rq: Root-mean-square roughness
SE: Spectroscopic ellipsometry
TCO: Transparent conductive oxide
TMA: Trimethylaluminum
XPS: X-ray photoelectron spectroscopy
XRD: X-ray diffraction
