# Supplementary material for: Effects of Al Doping on the Properties of ZnO Thin Films Deposited by Atomic Layer Deposition
Source: Nanoscale Res Lett. 2016 Sep 17;11:407. doi: 10.1186/s11671-016-1625-0 (PMC5026983; doi:10.1186/s11671-016-1625-0)
Supplement: Additional file 1: — Supporting information. (DOCX 298 KB) [file 11671_2016_1625_MOESM1_ESM.docx]

**Supporting Information**

1. The optical constants in Forouhi-Bloomer model are defined by [S1, S2]:

$$n\left( E \right)=n_{0}+\frac{XE+Y}{E^{2}-BE+C}$$

$$k\left( E \right)=A\left( E-E_{g} \right)^{2}/(E^{2}-BE+C)$$

Where

$$X=\left( \frac{A}{Q} \right)\left[ -\frac{B^{2}}{2}+E_{g}B-E_{g}^{2}+C \right]$$

$$Y=\left( \frac{A}{Q} \right)\left[ \frac{\left( E_{g}^{2}+C \right)B}{2}-{2E}_{g}C \right]$$

$$Q=(\frac{1}{2}){(4C-B^{2})}^{1/2}$$

Here *A*, *B* and *C* are fitting parameters related to the electronic configuration of the material, *E_g_* is the band gap energy and *n_0_* is the refractive index at high energy.

1. The fitting parameters from the Forouhi-Bloomer dispersion model.

| Samples | *n_0_* | *A* | *B* | *C* | *E_g_* (eV) | *RMSE* |
| --- | --- | --- | --- | --- | --- | --- |
| ZnO | 1.911 | 0.306 | 6.509 | 10.610 | 3.089 | 0.90 |
| AZO 50:1 | 1.907 | 0.304 | 6.529 | 10.678 | 3.092 | 0.91 |
| AZO 20:1 | 1.864 | 0.275 | 6.706 | 11.290 | 3.096 | 1.05 |
| AZO 10:1 | 1.839 | 0.240 | 7.055 | 12.531 | 3.102 | 1.01 |
| AZO 5:1 | 1.841 | 0.206 | 7.532 | 14.288 | 3.326 | 0.84 |

1. The SEM photograph of cross section of AZO 5:1 sample. The thickness of the film is 47.5 nm, which is close to the value of SE fitting.

**
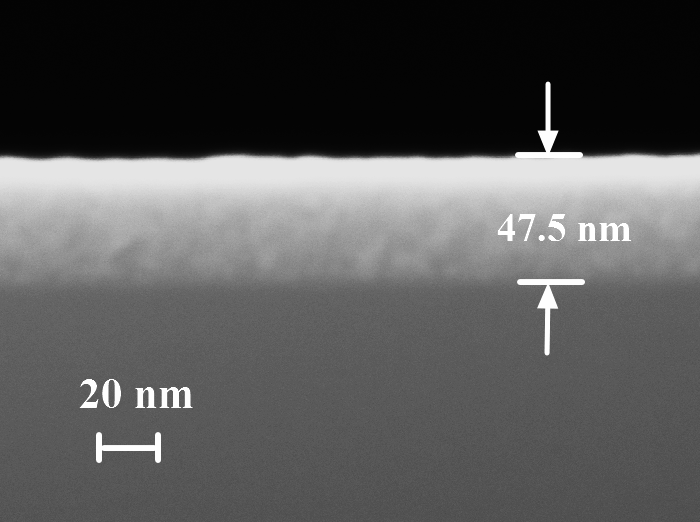
**

1. **The XPS survey spectrum of the AZO 5:1 films**


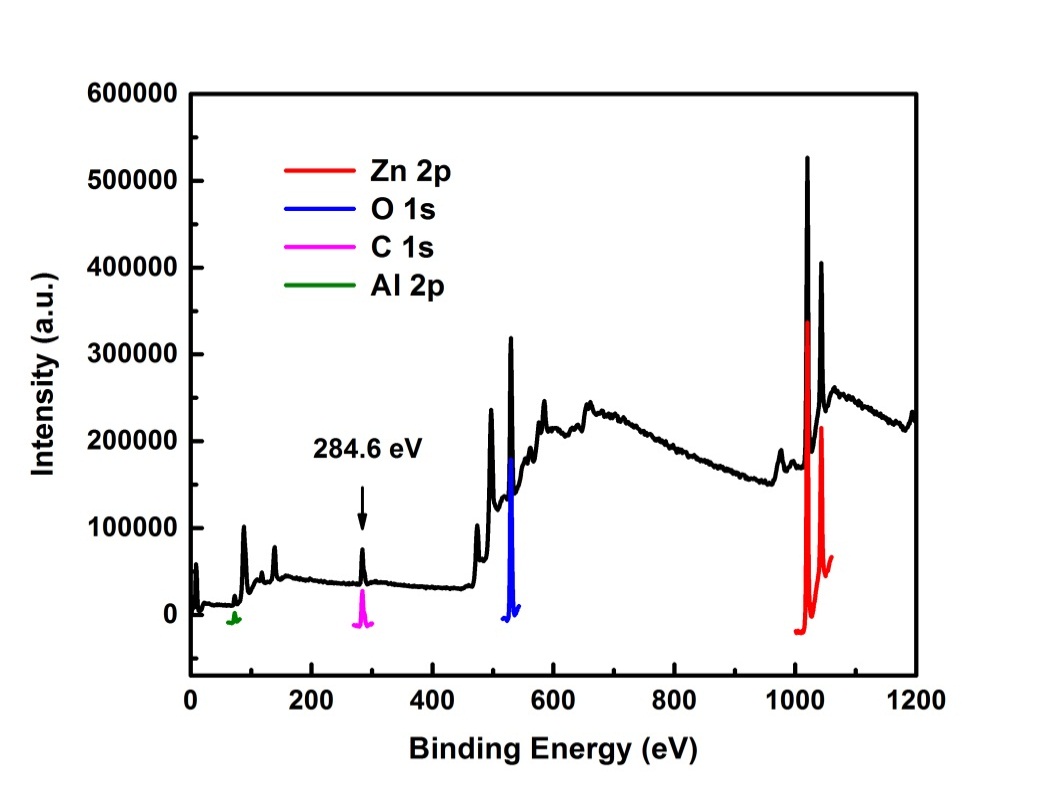


**References for supporting information**

S1. Forouhi AR, Bloomer I. Optical dispersion relations for amorphous semiconductors and amorphous dielectrics. Phys. Rev. B 1986; 34: 7018-7026.

S2. Forouhi AR, Bloomer I. Optical properties of crystalline semiconductors and dielectrics. Phys. Rev. B 1987; 38: 1865-1874.
